# Using Whole Exome Sequencing to Identify Genetic Causes of Neurodevelopmental Disorders in a Cohort of 11 Patients: A Single Center Experience

**DOI:** 10.3390/ijms262010176

**Published:** 2025-10-20

**Authors:** Marton Tompa, Gabriella Sinko, Judit Mally, Judit Karteszi, Bernadette Kalman

**Affiliations:** 1 National Genomic Center, Szentagothai Research Center, 20. Ifjusag Street, 7633 Pecs, Hungary; 2 Markusovszky University Teaching Hospital, Molecular Medicine, 5. Markusovszky Street, 9700 Szombathely, Hungary; gabriella@sinko.hu; 3 Neurorehabilitation, 3. Major Street, 9400. Sopron, Hungary; dr.habil.mallyjudit@gmail.com; 4 Genetic Counseling, Saint Rafael Hospital of Zala Castle County, 1. Zrinyi Street, 8900 Zalaegerszeg, Hungary; genetika.gen@zmkorhaz.hu; 5 Office of the Dean, School of Medicine, University of Pecs, 12. Szigeti Street, 7624 Pecs, Hungary

**Keywords:** neurodevelopmental disorder, whole exome sequencing, single nucleotide variants, copy number variants, molecular medicine

## Abstract

Neurodevelopmental disorders (NDDs) represent a heterogeneous group of diseases with a variety of clinical presentations related to different genetic, epigenetic, and environmental etiologies. Numerous pathogenic variants have been identified by comprehensive genetic approaches such as next-generation sequencing and chromosomal microarray analyses. This study included eleven pediatric patients with NDDs who were referred to our Molecular Medicine (MM) unit for further diagnostic workup. Whole exome sequencing (WES) was performed, and data were analyzed as part of a contracted service with the National Genomic Center and iBioScience LTD. Likely pathogenic single nucleotide variants in genes *DDX3X* c.869C>A, p.S290* and *CNOT1* c.920delG, p.G307Afs*32 in two patients, and pathogenic copy number variants in the 16p11.2 (16:29,690,418-30,200,285)x3 and 16p12.1-p11.2 (16:27,078,317-29,001,333)x3 regions in a third patient with NDDs were identified. In a fourth patient, the c.6839A>G, p.Gln2280Arg variant of uncertain significance was found in the *NIPBL* gene. Altogether, our study has revealed four novel variants in genes previously linked to NDDs. Identification of genetic causes of NDDs not only promotes establishing a more precise diagnosis and improves our understanding of disease pathogenesis but may also provide better means for developing preventive measures for the recurrence of this serious condition.

## 1. Introduction

According to the *Diagnostic and Statistical Manual of Mental Disorders*, *5th Edition* (DSM-V), the group of neurodevelopmental disorders (NDDs) includes autism spectrum disorders (ASDs), global developmental delay (GDD), attention deficit hyperactivity disorder (ADHD), specific learning disabilities, and motor and communication disorders [1,2]. NDDs, thus, represent a spectrum of overlapping phenotypes related to different etiologies involving genetic, epigenetic, and environmental factors. Even studying large cohorts, only a proportion of disease-causing genes and variants can be identified when using various approaches, including NDD gene panel and whole exome sequencing (WES), chromosomal microarray, and target gene, e.g., fragile X testing [2,3,4,5]. Heritability is estimated to be 90% for ASD and 40–55% for intellectual disability (ID) [2,6,7,8]. Monogenic forms may represent 25–50% of ID, GDD, and ASD [9].

In Hungary, chromosomal analysis and fragile X testing, both reimbursed by the national health insurance system (NHIS), are typically the first approaches in testing for a genetic etiology in NDDs. Analyses of copy number variations (CNVs) in 23 microdeletion regions by multiple ligation-dependent probe amplification (MLPA) are also performed for some patients. While internationally chromosomal microarray analysis is generally recommended as a first tier of testing [10,11,12,13], in Hungary, this test is partially covered by the NHIS based on case-by-case evaluation of expected outcome (i.e., therapeutic relevance). In most countries, second-tier testing for patients with NDDs (if the first-tier analyses were unrevealing) includes WES or intellectual disability (ID)/NDD panel analyses [2,11]. NHIS coverage for both WES and NDD panel testing falls under the same individual evaluation and selection procedures as chromosomal microarray studies in Hungary.

To promote advancements in molecular diagnostics, we have recently developed a new model involving a multidisciplinary collaboration among clinicians, clinical geneticists, molecular geneticists, and biologists, as well as bioinformatics scientists employed by the Molecular Medicine (MM) unit at the Markusovszky University Teaching Hospital (MUTH) and the National Genomic Center (NGC)/iBioScience Ltd. [14]. In the absence of uniform NHIS coverage, MUTH has set a generous internal fund aside for annually allowing 30–40 WES and Sanger studies. The main goal of this effort is to reduce diagnostic odyssey, refine disease subtype, facilitate personalized therapy, and support reproductive counseling for patients with rare and common genetically determined diseases seen at the MM of MUTH and tested at the NGC/iBioScience Ltd. In the last two and a half years, we have successfully identified pathogenic or likely pathogenic genetic variants in several diseases at our center [14,15,16,17]. From the small cohort of patients with NDDs, here we present those patients for whom WES identified novel pathogenic single nucleotide variants (SNVs) or CNVs.

## 2. Results

### 2.1. MN37

MN37 is a 10-year-old girl with intellectual disability, motor and speech developmental delay, and minor signs of dysmorphism. She was born from the first pregnancy at 40 weeks of gestation by cesarean section with an Apgar score of 9/10. She had an inguinal hernia operation at 4 months. She turned over at 3 months, raised her head at 4 months, crawled at 8 months, and stood up at 9 months but walked only with assistance until 16 months of age. Speech started at the age of 5 years and remained elementary until now. She went to nursery school at the age of 13 months. At the age of 2 years, she was examined for balance disorders and falls. A cranial MRI scan was performed at 2.5 years, where a smaller but regular vermis, narrow pons and brainstem, and dilatation of the occipital horn of the lateral ventricles were noted. EEG was normal. Since 2 years of age, she has been monitored by a pediatric neurologist for delayed psychomotor and speech development. She also has been examined by specialists from the Early Development Center and the Pedagogical Service Center in Budapest. Psychological assessments established her needs for special education, which she has been receiving with some benefits.

Family history: On the maternal side, the patient’s great-grandfather’s brother probably suffered from mental retardation and lived with his parents. The paternal grandmother started speaking late. Her father’s brother had epilepsy. The proband’s mother, father, and one younger sister are healthy.

On examination, she is a pleasant-looking girl of 10 years of age, and only some minor signs of dysmorphism are discernible: Wide nasal bridge, wide philtrum, narrow upper lip, ear cartilage of unusual shape, and long thin fingers. The left 5th finger is in a flexed position. Cranial nerves are intact; no paresis is noted in the extremities and deep tendon reflexes are brisk. Her gross spontaneous movements are somewhat dyscoordinated; fine movements such as writing and drawing are significantly affected. Speech is slurred and very rudimentary, saying only single words to express her basic needs, and usually with inappropriate grammar. She often talks about herself in the third person. Otherwise, she is alert, pays some attention to the examiner, and follows simple instructions but cannot count or read.

In the DNA of MN37, WES identified in a heterozygous state the NM_001356.5:c.869C>A, NP_001347.3:p.S290* likely pathogenic variant in the *DDX3X* gene (OMIM #300160) on chromosome X, position 41,203,496. This variant is absent from the gnomAD and the 1kG Phase 3 databases (PM2 moderate). It is a stop gain mutation with a loss of function (LoF) effect. LoF variants in *DDX3X* are known disease-causing mutations (PVS1 very strong). Based on the CADD score (7.59), the variant is damaging. The parents declined genetic testing for themselves since their second child showed normal development, and they did not plan to have more children.

### 2.2. MN90

MN90 is a 12-year-old boy with seizures, mild ID, motor and speech developmental delay, and autism. He is a child from an uncomplicated pregnancy, born at a gestational age of 38 weeks, weighing 2840 g, with an Apgar score of 8/10. His perinatal adaptation was uneventful. However, his motor and speech development was delayed. He started saying words at the age of 4–5 years. He currently attends a school for children with special needs. He sometimes has tantrums. His sleep is restless; he tosses and turns a lot. Enuresis frequently occurs. He has had seizures since the age of one and a half years. During infancy, his seizures were associated with respiratory arrest and cyanosis. Subsequently, the seizures presented with apnea and atonic state, mainly at night, during sleep, but occasionally also during daytime at school; when his eyes close, he freezes, stops all communication, becomes incontinent, and subsequently feels very sleepy. Usually, the convulsions last only a few minutes, during which no tongue biting occurs. EEG showed no clear epileptiform activity. His anti-seizure medication now includes levetiracetam and clobazam, and he receives diazepam suppositories as needed. According to the Pedagogical Assessment Service, he has mild ID and autism. One of his favorite occupations is playing video games and watching movies in English on his mobile phone. He learned English on his own. His cranial MRI showed a variation in the appearance of the corpus callosum, namely a narrowing in the boundary of the corpus and splenium, but without pathological changes in brain anatomy.

Family history: Mother and father are healthy. Mother had five pregnancies that resulted in only two live children (including the proband from the second pregnancy). Her first pregnancy was terminated due to an unknown genetic disorder. Another child was born at 24 weeks and lived only 3 days. The third lost child died of heart disease at 5 weeks of age. The only living younger brother suffers from ADHD. Also significant from the family history is that the mother has three healthy sisters. One sister has a little boy and a girl with autism and special educational needs. She also has two healthy older brothers. One of them has four children, two of whom are autistic. These family members live in another region of the country and could not be investigated at our center.

On examination, MN90’s cranium and vertebral column appear normal. His feet have slightly flattened arches, and his face shows minor signs of dysmorphism. Cranial nerves are normal. Muscle tone, mass, and strength are normal. Deep tendon reflexes are symmetric and 1+. No corticospinal signs are present. Sensorium appears normal for all qualities, as much as can be assessed in an autistic child. Coordination, standing, and walking are normal. Tandem walk and gait with closed eyes are normal. Speech is limited to single words, but there is no aphasia or dysarthria. He holds only brief eye contact. During the examination, he watches English language movies with full absorption on his mobile phone. He is vigilant, fully understands commands in Hungarian, and partially cooperates. He can also understand simple phrases and questions in English (no family member was aware of that previously) and occasionally responds in kind.

WES analysis identified in the *CNOT1* gene (OMIM #604917) a heterozygous dominant likely pathogenic variant NM_001265612.2:c.920delG, NP_001252541.1:p.G307Afs*32, located on chromosome 16, at position 58,616,973. The NM_001265612.2: c.920delG, p.(G307Afs*32) variant is missing from the gnomAD 2.1.1 and the 1kG Phase 3 databases (PM2 moderate). It is a null variant in a gene in which LoF variants are known to cause disease (PVS1 very strong). The younger brother of the proband with ADHD does not carry the variant. Second-degree relatives (cousins) with ASD could not be tested due to their remote location.

### 2.3. MN107

MN107 is a 7-year-old boy with somatic, speech, and cognitive developmental delay, minor signs of dysmorphism, and ADHD. He was born from a second pregnancy with an Apgar score of 10. His perinatal period as well as early development was uneventful. However, around 9 months of age, a global developmental delay was noticed. Somatic, motor, speech, and cognitive development were all affected. He also had a recurrent skin condition. For his walking difficulties, he received physiotherapy. Otherwise, he attended nursery school and then kindergarten.

Because of the persisting low weight for his age, comprehensive gastroenterological examinations were performed, but no absorption or metabolic abnormalities were found. A cranial MRI was without alterations. No cardiologic abnormalities were detected. His bone development appeared normal. His skin symptoms did not get worse upon exposure to sunlight and finally were described as eczema. A psychiatric examination concluded that he had ADHD, for which special education was recommended. He currently rides horses, attends speech therapy, and takes part in a special education program.

Family history is negative for neurological and psychiatric disorders on both the maternal and paternal sides. The proband’s older brother is currently 10 years old and healthy.

On examination, MN107’s status is significant for nanosomy, microcephaly, and minor dysmorphic signs, including a small jaw and slightly broad nasal bridge. He has dry skin with spotty signs of eczema. The cranium and vertebral column are normal. Cranial nerves are without abnormalities. Slightly hypotonic skeletal muscles with retained strength and symmetric 1+ deep tendon reflexes were noted as well as hyperextension in the knees. Coordination in the extremities is normal. Standing and walking are fine. Tandem gait and walking with closed eyes are normal. No sensory abnormalities can be detected. He speaks a lot, but with errors of articulation and grammar. He is very vigilant, but his attention can be retained only for short periods of time. He understands and mostly follows instructions and has a friendly disposition, but interpersonal communication is limited due to his scattered attention.

**Figure 1:**
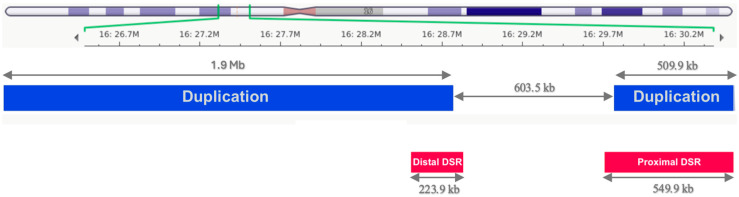
The detected CNVs in MN107 overlap with the 16p11.2 dose-sensitive region.

In connection with the phenotype, WES revealed two heterozygous copy number variants (CNVs) within the 16p11.2 (16:29,690,418-30,200,285)x3 and the 16p12.1-p11.2 (16:27,078,317-29,001,333)x3 chromosomal regions. The size of the former duplication CNV is 509.9 kb, and of the latter duplication CNV is 1.9 Mb (Figure 1). Evidence supports the pathogenic role of the duplication in the proximal and distal regions. The proximal region includes 29 genes (*TBX6*, *QPRT*, *C16orf54*, *ZG16*, *KIF22*, *MAZ*, *PRRT2*, *PAGR1*, *MVP*, *CDIPT*, *CDIPTOSP*, *SEZ6L2*, *ASPHD1*, *KCTD13*, *TMEM219*, *TAOK2*, *HIRIP3*, *INO80E*, *DOC2A*, *C16orf92*, *TLCD3B*, *LOC112694756*, *ALDOA*, *PPP4C*, *YPEL3*, *YPEL3-DT*, *GDPD3*, *MAPK3*, *CORO1A*), of which *TBX6* is a driver gene. The distal region includes 41 genes (*IL21R KATNIP*, *CLN3*, *TUFM*, *ATP2A1*, *CD19*, *LAT*, *KDM8*, *NSMCE1*, *IL4R*, *GTF3C1*, *GSG1L*, *XPO6*, *SBK1*, *APOBR*, *IL27*, *NUPR1*, *SGF29*, *ATXN2L*, *SH2B1*, *RABEP2*, *NFATC2IP*, *SPNS1*, *C16orf82*, *NPIPB6*, *EIF3CL*, *NPIPB7*, *NPIPB8*, *LINC02129*, *NSMCE1-DT*, *IL21R-AS1*, *LOC100128079*, *MIR6862-1*, *SULT1A2*, *SULT1A1*, *EIF3C*, *MIR6862-2*, *NPIPB9*, *MIR4721*, *ATP2A1-AS1*, *MIR4517*), of which *SH2B1* is a driver gene.

There is a known 549.9 kb proximal and a 223.9 kb distal dose-sensitive region within chromosome 16p11.2. The CNV within the 16p11.2 region (OMIM #614671) has autosomal dominant inheritance or may occur de novo. The associated phenotype has been described as duplication syndrome of 16p11.2 (OMIM #614671). The 509.9 kb duplication of MN107 overlaps with the proximal duplication, while the 1.9 Mb duplication overlaps with the distal dose-sensitive region (Figure 1). Ample literature supports that duplication events in the proximal region are associated with speech, linguistic, and motor developmental delays and ASD, intellectual disability, ADHD, seizures, microcephaly, and reduced body mass index [18,19,20] (5H). The literature supporting the pathogenic role of the distal CNV is sparser but shows association with similar phenotypic signs, including microcephaly, low body mass index, scoliosis, ASD, ADHD, and abnormalities in the brain (5H) [21,22,23]. Both the proximal and the distal region CNVs overlap with a previously reported dosage-sensitive, triplosensitive gene or genomic region, which is known to cause a clinical phenotype when altered in copy number (A2). Also characteristic for both regions is the reduced penetrance. In MN107, we confirmed the two CNVs detected by WES by performing and analyzing the data of whole genome sequencing. The parents declined genetic testing for themselves, as they do not plan to have more children. Testing for the healthy older brother may be considered when he reaches adult age.

Figure 1 depicts the two duplication CNVs in MN107. In the lower part of the figure, the 16p11.2 dosage-sensitive regions (DSRs) are highlighted in red (the distal region on the left is 223.9 kb, and the proximal region on the right is 549.9 kb in size). In the middle part of the figure, the CNVs identified in MN107 are shown in blue (the distal CNV on the left is 1.9 Mb, and the proximal CNV on the right is 509.9 kb in size). The affected area of chromosome 16 is indicated in the uppermost part of the figure.

### 2.4. MN126

MN126 is a 16-year-old girl with intellectual disability, dysmorphisms, and a history of seizures. Her perinatal history was normal, but some dysmorphic anomalies were already noted in her early age. Between 4 and 13 years of age, she had epilepsy treated with valproic acid. However, she has been lately free of seizures without antiepileptic treatment. A cranial MRI showed no alterations, but an X-ray of the spinal cord revealed a bone abnormality in the Th6 vertebra. During her early teenage years, asymmetric development of her breasts was noted, and she also had significant temporary hair loss, but no endocrine abnormalities were identified. Due to her intellectual disability, she has been tested by the Early Development and the Pedagogical Service Center, which revealed an IQ of 58 at the age of 12 years.

Family history is significant on the father’s side, as the paternal grandmother suffered from schizophrenia, and a paternal aunt had an intellectual disability. The parents are healthy and divorced. The proband had no sisters or brothers at the time of study.

On examination, MN126 appears to have short stature, microcephaly, low anterior and posterior hairline, dysmorphic face with thick, arched eyebrows, close-set eyes, gothic palate, and small hands and feet. On her back, hypertrichosis is noted. Her breasts appear asymmetrical, the right one being smaller and showing different morphology than the left. Cranial nerves are without alteration. Muscle tone, mass, and strength are normal. Deep tendon reflexes are symmetrical. While Hofmann–Trömner signs are positive in both hands, the Babinski sign cannot be elicited. No sensory abnormalities or appendicular or axial ataxia are seen, but her movements are a bit disharmonic. While she has no aphasia or dysarthria, communication is somewhat difficult, and she expresses herself in simple sentences. She is very alert but shy. Cognitively she is behind her age and attends a special program for children with ID. Her attention can be easily aroused and directed, and she readily follows simple commands. Reading is possible, but arithmetic skills are quite limited. She can write down her name and address.

Whole exome sequencing revealed a heterozygous SNV in the *NIPBL* gene (OMIM #608667) NM_133433.4:c.6839A>G, NP_597677.2:p.Gln2280Arg (Revel: 0.967, Alpha missense score: 0.9955). This variant has no ClinVar or LOVD documentation. Its manual interpretation is a variant of uncertain significance (VUS). The variant is missing from the gnomAD 2.1.1 and the 1kG Phase 3 databases (PM2 moderate), is located in a well-characterized, functionally important domain [24] (PM1 supporting), has multiple in silico prediction tools that support that the variant is located at a conserved position, the amino acid substitution may be functionally significant (PP3 supporting), and the patient’s phenotype is specific to the disease (PP4 supporting). Pathogenic variants in the *NIPBL* gene account for most cases of the rare developmental disorder, Cornelia de Lange syndrome (CDLS) (OMIM #122470). Unfortunately, the biological parents were unavailable for their genetic testing. The Face2Gene phenotyping software (https://www.face2gene.com/) indicated Cornelia de Lange syndrome as a first possibility with high probability based on the patient’s clinical appearance.

Table 1 outlines the main clinical characteristics and genotypes of the four patients with NDDs.

Table 2 summarizes the in silico prediction tools employed for variant effect prediction. MSA-SIFT and MSA-PolyPhen2 assess the functional impact of amino acid substitutions using multiple sequence alignments. PhyloP and GERP++ measure nucleotide conservation to identify constrained sites. CADD integrates diverse annotations to score variant deleteriousness. REVEL combines multiple predictors for missense pathogenicity, while AlphaMissense uses deep learning with AlphaFold-based structural context.

## 3. Discussion

NDD/ID spectrum disorders represent a very heterogeneous group of diseases regarding phenotypic presentation, comorbidities, and etiology. Even those forms with genetic etiology have more than 1500 known primary ID genes predominantly affecting chromosome organization, cell cycle, gene expression and metabolism, mechanisms that influence neurogenesis, excitatory–inhibitory neuron development, and synaptic organization in the very early stages of fetal brain development [25,26,27,28]. Several databases and gene lists have been generated, and overviews of the databases have been reported [28]. With the advent of modern sequencing technologies and innovative model systems, the discovery and confirmation of novel genes and variants in various phenotypic subtypes of NDDs are continuously advancing [29,30,31,32]. In a timely topical review of molecular and genetic mechanisms of ASD [33], the authors presented relative risks and biological effects of rare SNV and CNV variants contributing to the disease by affecting gene expression regulation, neuronal communication, and cytoskeleton integrity. Most recent studies complement the catalogue of previously identified missense, truncating, and splice site SNVs or CNVs within protein-coding genes by revealing novel variants in non-protein-coding genes. Modification of the RNA methylome by mutations in effector protein genes is also of interest, because it disrupts translation dynamics, synaptic function, and energy production, or causes cellular stress in NDDs [34]. Analyses of R-loop-forming regions not only opened a new window for disease mechanisms but also identified genetic etiology in a large proportion of patients negative for causative mutations in protein-coding genes. R-loops are DNA–RNA hybrids that form at sites of active transcription [35,36,37]. An excess of de novo variants in R-loop regions and the enrichment of these variants in ribosome, small nucleolar RNA, and small nuclear RNA genes were discovered in rare disease cohorts [36,37]. Rare disease-causing variants in the spliceosomal RNA-encoding genes, *RNU2-2* and *RNU5B-1*, were reported following the discovery of a heterozygous pathogenic variant in the U4 small nuclear RNA, *RNU4-2*, in patients with NDD [36,37]. The above highlights of existing data reflect the continuous progress in research and the genetic complexity of NDDs.

The combined prevalence of NDD/ID was estimated to be 17.8% between 2015 and 2017 in the USA [2,27]. The European Reference Network (ERN) Intellectual disability, TeleHealth, Autism, and Congenital Anomalies (ITHACA)1 is a virtual expertise network that recently established priority-setting criteria for etiology-specific clinical practice guidelines (CPGs) [27]. This study emphasizes the high burden of NDD/ID for affected individuals as well as for family and society. Early diagnosis and multidisciplinary intervention are essential to reduce disability and costs. Identification of genetic etiology even in the clinical setting is of high priority in order to diminish risks for familial recurrence. While ADHD and ASD have been extensively studied [38,39], genetic data are relatively sparse for NDDs in Hungary [39,40].

Our multidisciplinary program of MM at MUTH, in contract with the NGC/iBioScience Ltd., started its operation at the end of 2022. Until the end of July 2025, 63 probands with a variety of clinical conditions were studied by WES, not including here the asymptomatic parents from trio analyses and other family members subjected to Sanger sequencing for segregation analyses. Of the 63 patients, 11 were pediatric cases diagnosed with some forms of NDD/ID. Table 1 shows phenotypic and genetic data of those three patients with NDD/ID, for whom WES revealed likely pathogenic or pathogenic variants, and of one additional patient whose NDD-relevant variant was classified as VUS. Table 2 summarizes the in silico prediction results for the missense and truncating variants and lists each applied algorithm with the corresponding scores. The four presented variants are novel, absent from the ClinVar, gnomAD, and 1kG databases; therefore, they may further broaden knowledge regarding the genetic etiology and mechanisms of these severe disorders.

The first presented female patient, MN37, harbors a heterozygous *DDX3X* likely pathogenic nonsense variant appearing causative for her phenotype, compatible with the DDX3X syndrome. Levy et al. [41] reviewed this entity, an X-linked monogenic neurodevelopmental disorder affecting predominantly (but not exclusively) female patients with ID, ASD, language delays, ADHD, brain MRI abnormalities, and other medical problems. At the time of the review, two hundred patients with DDX3X syndrome had been reported, showing varying complexity of presentations and symptoms in the neurological, psychiatric, ophthalmologic, and gastrointestinal areas. The prevalence of DDX3X syndrome was estimated to be between 1% and 3% in females whose NDD symptoms could not be explained by other etiologies. The p.S290* variant detected in MN37 is absent from the list of previously reported pathogenic SNVs and is located in the helicase ATP-binding domain of *DDX3X*, a hot spot for pathogenic missense and truncating mutations. Our patient represents yet another case of DDX3X syndrome with a new mutation.

*DDX3X* (OMIM #300160) encodes an evolutionarily conserved DEAD-box RNA helicase involved in a broad range of processes from RNA biogenesis to decay and influences RNA splicing, transcription, and transport. Through these mechanisms this gene regulates cell cycle, apoptosis, and WNT signaling, thereby participating in embryonic and neuronal development. The DEAD-box naming stemmed from the Asp–Glu–Ala–Asp (D–E–A–D) motif present in RNA helicases [42]. DDX3X has three parts, including an N-terminal extension, a helicase core, and a C-terminal extension. The helicase core, where the protein-truncating variant of MN37 is located, has 12 conserved motifs, many of which are involved in ATP binding [43]. ATP binding is needed for helicases to adopt a certain conformation that allows hydrolysis and couples ATP binding to RNA binding and unwinding, thereby affecting RNA metabolism. RNA helicases are extensively studied, but their cofactors, substrates, post-translational modifications, protein–protein interactions, or their roles in signaling are only partially understood [42]. The DDX3 (DDX3X/DDX3Y) helicases have been most intensively investigated for their roles in immune response regulation, anti-viral defense, and cellular stress responses [44,45]. The roles of *DDX3X* variants defining the unique entity of DDX3X syndrome within the NDD/ID spectrum disorders were also examined [46,47]. The mutational landscape of *DDX3X* is well established [41]. Phenotype and genotype correlation analyses showed that individuals with protein-truncating (nonsense, frameshift, splice site) variants generally had less severe clinical presentations than those with missense variants [41,48]. Longitudinal clinical data of our MN37 patient with a protein-truncating variant are compatible with this observation, as her language skills, academic advancement, and behavioral characteristics indeed somewhat improved over time due to a complex, personalized, multifaceted program.

The clinical presentation of our second case, MN90, predominantly shows signs of motor and speech developmental delay, seizures, ID, ASD, and behavioral problems, in association with a heterozygous truncating variant in the *CNOT1* gene (OMIM #604917), classified as likely pathogenic.

ASD is, unfortunately, not a rare disease: 1 in 44 children was estimated to have the diagnosis in 2021 in the USA, and the numbers are still growing [49]. This disabling condition with varying severity causes difficulties in social interactions and verbal and nonverbal communication, while also involving other behavioral problems and comorbidities. Boys are affected four times more frequently than girls. ASD may have monogenic etiology in about 5% of cases. Typically, however, ASD is a complex trait disorder with multiple risk variants, each exerting a small effect, but in interaction with each other and with environmental factors. Genetic factors contribute 40–80% risk to the disease. Environmental contributors include older parental age, intrauterine and perinatal complications, and many other less defined factors. A constellation of inherited and acquired factors may result in the ASD phenotype through impairing brain development, neuronal growth, and synaptic organization [50]. Epigenetic regulation during early brain development and even at the single-cell level has been recently explored [49,50].

In MN90, we identified a heterozygous likely pathogenic protein-truncating variant in the *CNOT1* gene (c.920delG, p.G307Afs*32). This gene encodes a subunit of the CCR4-NOT complex involved in mRNA stability and post-transcriptional regulation [51]. CNOT1 is the largest subunit of the complex and has five domains called HEAT, TTP, CAF1, DUF3819, and Not1. CNOT1 acts as a scaffold protein [51]. However, it is also involved in driving mRNA deadenylation, thereby shortening the poly(A) tail in mRNA and leading to its degradation. Through this function, CNOT1 contributes to transcriptional repression affecting early embryonic development [51,52]. Monoallelic missense *CNOT1* variants have been associated with holoprosencephaly-12 with pancreatic agenesis (OMIM #619033) [52,53]. Monoallelic nonsense, missense, and splice site variants of *CONT1* were recently associated with the Vissers-Bodmer syndrome (OMIM #604917) [52,54]. Available evidence has established a definite gene–disease relationship [52,54]. The Vissers-Bodmer syndrome may involve motor and speech developmental delay, ID, epilepsy, hypotonia, dysmorphism, ASD, and behavioral problems such as ADHD. The p.G307Afs*32 *CNOT1* variant is a protein-truncating variant resulting in the elimination of all important functional domains by the early stop codon. The phenotypic features of MN90 greatly overlap with those of Vissers-Bodmer syndrome. Of note, a recently reported patient with another early frameshift variant (p.Gly172Alafs*5) that resulted in protein truncation had somewhat different phenotypic features such as short stature, growth hormone deficiency, spina bifida, and horseshoe kidney in addition to mental retardation and ADHD, features only partially overlapping with the presentations of our patient and reflecting phenotypic variability of the disease [52]. Currently available data, however, suggest no major phenotypic differences in patients with loss of function or missense variants possibly related to shared pathogenic mechanisms [52,55]. Our phenotypic and genotypic observations in MN90 extend knowledge about the recently described Visser-Bodmer syndrome. Nevertheless, the interpretation of the novel *CNOT1* c.920delG, p.G307Afs*32 variant is not unequivocal. Its clinical significance cannot be presently supported by data in the literature, and functional research is needed to gain further support for its pathogenicity.

The phenotype of our third case, MN107, was associated with structural variations within the 16p11.2 (16:29,690,418-30,200,285)x3 and the 16p12.1-p11.2 (16:27,078,317- 29,001,333)x3 chromosomal regions (Figure 1). Structural alterations within the chromosome 16p11.2 region are associated with autosomal-dominant or de novo-occurring 16p11.2 duplication syndrome (OMIM #614671) [56]. Chromosome region 16p11.2 contains a 549.9 kb proximal and a 223.9 kb distal dose-sensitive region. The 509.9 kb duplication event identified during our study overlaps with the proximal dose-sensitive region, while the 1.9 Mb duplication event overlaps with the distal dose-sensitive region. Duplication CNVs partially or completely overlapping with the two regions have diverse phenotypic presentations.

Ample amount of evidence supports the pathogenic role of the duplication in the proximal region encompassing 29 genes, including *TBX6* as a driver gene (see detailed list of genes in the Results section). The *TBX6* product, T-Box transcription factor, is needed for Mesp2 expression during somitogenesis in mice [57]. TBX6 also regulates SOX2, which plays important roles in early embryonic development, including neural tube formation [58]. The number of independent studies on this CNV in the literature is relatively high, with documented phenotypic manifestations consistent with speech, language, and motor developmental delay and ID, congenital developmental disorders, ASD, behavioral conduct disorders (including ADHD), epileptic seizures, microcephaly, and reduced body mass index (BMI) [18,19,20]. Interestingly, deletions in this region have been associated with obesity [59]. The phenotypes (small BMI or obesity) have been correlated with the direction of changes at transcript levels for genes mapping within the region. The reciprocal impact of these 16p11.2 CNVs suggests that being severely obese or underweight could have mirroring etiologies, related to expression of genes within the 16p11.2 region. There is limited evidence to support the pathogenic role of the duplication in the distal region encompassing 41 genes (see Result section), but diverse and relatively nonspecific clinical phenotypes, including reduced microcephaly, low BMI, scoliosis, ASD, ADHD, and some brain morphological abnormalities, have also been described [21,22,23]. CNVs in both regions are characterized by reduced penetrance and often occur de novo.

In the fourth patient, MN126, WES analysis identified a heterozygous missense variant, NM_133433.4:c.6839A>G, NP_597677.2:p.Gln2280Arg, in the *NIPBL* gene, which has not been previously reported and is missing from the ClinVar and LOVD databases. Based on manual interpretation, the variant’s current classification status is VUS. It is located in the evolutionarily conserved Nipped-B-C domain of the gene. CDLS is a genetically heterogeneous disorder with overlapping clinical presentations and varying severity [60,61]. Based on genetic etiology, six subtypes (CDLS1-CDLS6, OMIM # 122470, 300590, 610759, 614701, 300882, 620568) are distinguished. CDLS1 represents 50–60% of the entity associated with pathogenic missense and protein-truncating variants as well as chromosomal structural variants affecting the *NIPBL* gene [62,63]. CDLS presents with multisystem abnormalities, including facial dysmorphisms with low anterior hairline, arched eyebrows, anteverted nares, long philtrum, maxillary prognathism, thin lips, and ‘carp’ mouth, growth retardation, bony abnormalities in the hands, and intellectual disability. The phenotypic presentation of MN126 is compatible with the reported features of CDLS, including the low stature, low anterior and posterior hairline, strong arched eyebrows and gothic palate, small hands, gross breast asymmetry, significant intellectual disability, limited verbal communication, and hypertrichosis on the back. The detection of a heterozygous missense variant in the *NIPBL* gene refines the diagnosis as CDLS1 in our patient. The probability of CDLS is also strongly supported by Face2Gene phenotyping. Most such cases in the literature are reported as sporadic, similar to the case of MN126, in whose family no similar phenotype was noted. The NIPPED-B-LIKE, or *NIPBL* (OMIM #608667), gene product forms a dimer with MAU2 (614560) that facilitates loading the cohesin complex onto sister chromatids [63,64]. These authors demonstrated that direct interactions with transcription factors lead to the localization of the cohesin-loader complex NIPBL/MAU2 within enhancers and promoters and facilitate the forming of enhancer-promoter loops. The cohesin complex is also involved in the regulation of chromatid separation during mitosis and meiosis, repair of DNA double-strand breaks, and transcription. The Nipped-B-C domain of the gene is close to the C terminus of the protein, the site where the variant in MN126 is located. This domain is also called the “sister chromatid cohesion C-terminus” of NIPLB, involved in protein–protein interactions, cohesion loading or cofactor binding, and in the recruitment of acetylases to mediate chromatin modification [65]. Through these mechanisms, NIPBL plays important roles during embryonic development and significantly affects brain development [66]. More recently, the question has been raised if the underlying molecular mechanism of CDLS may also be involved in cancer predisposition [67]. The clinical significance of the variant detected in MN126 is pending upon further confirmation.

While many subtypes of neurodevelopmental disorders may be rare, NDDs as a group represent not an infrequent condition. Defining the genetic etiology of these severe clinical conditions facilitates establishing the precise diagnosis and contributes to a better understanding of the complex pathogenesis. Through our four cases, we have extended the phenotypic spectra of the DDX3X, Vissers-Bodmer, 16p11.2 duplication, and Cornelia de Lange syndromes, while also adding novel genetic variants to the lists of known mutations. A limitation of our presentation is that only singletons were studied in WES since parents were unavailable for trio analyses, thereby preventing the determination of inheritance or de novo occurrence of variants. Nevertheless, through the small success of the initial work at our recently established MM center, we laid the ground for a more comprehensive mapping of causative genetic variants of NDDs in our region, which will complement information in international databases. The four variants reported here have been submitted to ClinVar and will be publicly visible after the publication of this paper.

## 4. Materials and Methods

### 4.1. Patients

Altogether, we tested 11 pediatric patients with NDDs, having some degrees of intellectual disability and various combinations of dysmorphism, somatic, motor, and cognitive delay, complex developmental delay, ASD, ADHD, and other behavioral abnormalities. Of these eleven patients, genetic etiology could be identified for four children. All four patients were studied as singletons, as either parents declined to participate or extended resources for their WES were unavailable. All but one patient were intra-institutionally referred to our MM unit from the Department of Pediatrics, MUTH. A single patient came from the care of the Neurorehabilitation Center, Sopron. The age range at the time of initiation of the diagnostic workup was 2–16 years. Eight boys and 3 girls were included. The main purpose of the genetic analyses was to define the molecular genetic determinants of the clinical phenotypes. Parents received pre-test genetic counseling, and after their written consent, 5–6 mL of blood was drawn from each patient. Post-test counseling was also provided. This study was conducted in accordance with the Declaration of Helsinki and approved by the Institutional Review Board, Markusovszky University Teaching Hospital.

### 4.2. Laboratory Methods

DNA was extracted from EDTA-treated peripheral blood by using the QIAamp^®^ DNA Mini Kit (Qiagen, Hilden, Germany) at the MM, MUTH. The subsequent laboratory procedures were performed at the NGC. For whole exome sequencing, libraries were prepared using the xGen DNA Library Prep Kit (Integrated DNA Technologies Inc. [IDT], Coralville, IA, USA). In brief, DNA was fragmented by enzymatic treatment, and after adapter ligation, libraries were PCR amplified. Exome capture was performed by using the xGen Exome v2 Panel probes (IDT). After quality check by the TapeStation 4200 (Agilent Technologies, Santa Clara, CA, USA), libraries were also quantified by Qubit 3.0 (Invitrogen, Waltham, MA, USA) and then sequenced on a NovaSeq 6000 machine (Illumina Inc., San Diego, CA, USA) with 2 × 150 paired-end reads.

### 4.3. Bioinformatic Analyses

Bioinformatic analyses were carried out by the team of MM, MUTH, and iBioScience LTD. Raw sequencing data were generated with bcl2fastq (v2.20.0.422). SNVs and small INDELs (<50 bp) were identified using a custom pipeline. Raw reads were quality-checked with FastQC (v0.11.9) and trimmed with fastp (v0.21.0). High-quality reads were aligned to hg19/GRCh37 using BWA-MEM (v0.7.17), and BAM files were processed with Picard Tools (v2.23.3) for sorting, indexing, duplicate removal, and metadata addition. Mapping metrics were assessed with Picard and Qualimap (v2.2.1). Base quality recalibration was performed with GATK BSQR (v4.5.0.0) using dbSNP155, followed by variant calling and filtering with GATK modules. Variant annotation and interpretation were performed with VarSeq 2.6.2 (Golden Helix Inc., Bozeman, MT 59715, USA), using RefSeq, gnomAD, dbSNP, NHLBI, ClinVar, OMIM, and in silico predictors (PolyPhen-2, SIFT, PhyloP, GERP++, CADD, REVEL, Splice AI), along with the current literature. Genomic variants were classified per ACMG/ACGS 2024 guidelines [68].

## 5. Conclusions

We presented four pediatric patients with NDDs associated with novel genetic variants of pathogenic, likely pathogenic, and uncertain significance. These patients were studied at our recently established MM at MUTH in contract with the NGC/iBioScience LTD services. In the routine diagnostic setting, searching for causative genetic variants of NDDs is increasingly, but not yet uniformly, feasible. Prevalence of NDDs is still high, and the disease is a serious condition with a significant burden for the patient, family, and society. Refinements of the genetic landscape may not only facilitate a better understanding of the heterogeneous disease pathogenesis but can also help prevent recurrence by genetic counseling.

## Figures and Tables

**Table 1:** Summary of phenotypic and genotypic features of the four patients.

Proband	MN37	MN90	MN107	MN126
Age of disease onset (years)	1	1	1	congenital
Age at study (years)	10	12	7	16
History of hypotonia	no	no	yes	no
Motor delay	yes	yes	yes	no
Speech delay	yes	yes	yes	yes
Somatic delay	no	no	low weight	no
Seizures	no	yes	no	yes
ID	yes	yes	yes	yes
ASD	no	yes	no	no
ADHD	no	no	yes	no
Behavior problems	yes	yes	yes	no
Dysmorphism	minor	minor	minor	moderate
Other	no	no	eczema	asymmetric breasts
MRI abnormality	narrow pons and brainstem, dilatation of occipital horns of lateral ventricules	normal anatomical variation	no	no
Positive family history of NDDs	yes, but remote	younger brother ADHD, 4 cousins on mother’s side have ASD	no	paternal aunt had ID
Previous genetic tests	none	46XY kariotype, *FMR1* normal alleles	46XY kariotype	46XX karyotype, array CGH planned, but not completed
Gene	*DDX3X*	*CNOT1*	See details in Results	*NIPBL*
SNV	NM_001356.5: c.869C>A, NP_001347.3: p.S290*	NM_001265612.2: c.920delG, NP_001252541.1: p.G307Afs*32	no	NM_133433.4: c.6839A>G, NP_597677.2: p.Gln2280Arg
CNV	no	no	16p11.2 (16:29,690,418-30,200,285)x3 and 16p12.1-p11.2 (16:27,078,317-29,001,333)x3	no
Variant classification	LP	LP	P	VUS
Novel	yes	yes	yes	yes
Established diagnosis	DDX3X syndrome	Vissers-Bodmer syndrome	chromosome 16p11.2 duplication syndrome	Cornelia de Lange syndrome

**Table 2:** Summary of applied in silico prediction tools.

Genes	*DDX3X*	*CNOT1*	*NIPBL*
Variants	NM_001356.5:c.869C>A NP_001347.3: p.S290*	NM_001265612.2:c.920delG NP_001252541.1:p.G307Afs*32	NM_133433.4:c.6839A>G NP_597677.2:p.Gln2280Arg
MSA-SIFT	-	-	1.00
MSA-PolyPhen2	-	-	1.000
PhyloP	7.77	7.80	8.96
GERP++	19.50	19.56	18.56
Combined Annotation-Dependent Depletion (CADD)	9.67	4.65	5.33
Revel	-	-	0.97
Alpha Missense	-	-	0.996

## Data Availability

Data are unavailable due to privacy or ethical restrictions.

## Abbreviations

NDD	Neurodevelopmental delay
WES	Whole exome sequencing
DSM-V	*Diagnostic and Statistical Manual of Mental Disorders*, *5th Edition*
ASDs	autism spectrum disorders
GDD	global developmental delay
ID	intellectual disability
NHIS	national health insurance system
CNVs	copy number variations
MLPA	multiple ligation-dependent probe amplification
MM	Molecular Medicine
MUTH	Markusovszky University Teaching Hospital
NGC	National Genomic Center
SNV	single nucleotide variant
CDLS	Cornelia de Lange syndrome
ERN	The European Reference Network
ITHACA	Intellectual disability, TeleHealth, Autism, and Congenital Anomalies
CPG	clinical practice guideline
ADHD	Attention Deficit Hyperactivity Disorder
